# Doxorubicin‐induced cardiotoxicity is maturation dependent due to the shift from topoisomerase IIα to IIβ in human stem cell derived cardiomyocytes

**DOI:** 10.1111/jcmm.14346

**Published:** 2019-05-20

**Authors:** Ning Cui, Fujian Wu, Wen‐Jing Lu, Rui Bai, Bingbing Ke, Taoyan Liu, Lei Li, Feng Lan, Ming Cui

**Affiliations:** ^1^ Department of Cardiology Peking University Third Hospital Beijing China; ^2^ Department of Respiration The Affiliated Hospital of Qingdao University Qingdao China; ^3^ Beijing Lab for Cardiovascular Precision Medicine Anzhen Hospital, Capital Medical University Beijing China

**Keywords:** cardiomyocyte, cardiotoxicity, doxorubicin, maturity, pluripotent stem cell, topoisomerase

## Abstract

Doxorubicin (DOX) is widely used to treat various cancers affecting adults and children; however, its clinical application is limited by its cardiotoxicity. Previous studies have shown that children are more susceptible to the cardiotoxic effects of DOX than adults, which may be related to different maturity levels of cardiomyocyte, but the underlying mechanisms are not fully understood. Moreover, researchers investigating DOX‐induced cardiotoxicity caused by human‐induced pluripotent stem cell‐derived cardiomyocytes (hiPSC‐CMs) have shown that dexrazoxane, the recognized cardioprotective drug for treating DOX‐induced cardiotoxicity, does not alleviate the toxicity of DOX on hiPSC‐CMs cultured for 30 days. We have suggested that this may be ascribed to the immaturity of the 30 days hiPSC‐CMs. In this study, we investigated the mechanisms of DOX induced cardiotoxicity in cardiomyocytes of different maturity. We selected 30‐day‐old and 60‐day‐old hiPSC‐CMs (day 30 and day 60 groups), which we term ‘immature’ and ‘relatively mature’ hiPSC‐CMs, respectively. The day 30 CMs were found to be more susceptible to DOX than the day 60 CMs. DOX leads to more ROS (reactive oxygen species) production in the day 60 CMs than in the relatively immature group due to increased mitochondria number. Moreover, the day 60 CMs mainly expressed topoisomerase IIβ presented less severe DNA damage, whereas the day 30 CMs dominantly expressed topoisomerase IIα exhibited much more severe DNA damage. These results suggest that immature cardiomyocytes are more sensitive to DOX as a result of a higher concentration of topoisomerase IIα, which leads to more DNA damage.

## 1 INTRODUCTION

Doxorubicin (DOX), one of the anthracyclines, is widely used to treat various cancers in adults and children, including leukaemias, lymphomas and solid tumours.^1^, ^2^, ^3^, ^4^ However, DOX‐induced cardiotoxicity, characterized by dilated cardiomyopathy and heart failure, limits its clinical application.^5^, ^6^, ^7^ Furthermore, clinical studies have shown that DOX‐induced cardiotoxicity is age‐related. Children are more susceptible to the cardiotoxic effects of DOX than adults, especially children younger than 4 years.^8^, ^9^, ^10^, ^11^, ^12^ This may be due to the level of maturity of cardiomyocyte. It has been shown in previous studies that DOX‐induced apoptosis gradually decreases during cell maturation.^13^, ^14^, ^15^ However, the underlying mechanism remains unclear due to a lack of in vitro cellular maturation models.

Since human induced pluripotent stem cell‐derived cardiomyocytes (hiPSC‐CMs) can mimic many aspects of human cardiomyocytes, such as contractile function, cardiac genes expression and electrophysiological phenotypes, they have become an important technology in human cardiovascular disease modelling.^16^ Moreover, hiPSC‐CMs provide a drug‐testing platform for large‐scale screening of compounds. The US Food and Drug Administration initiated a new program, the Comprehensive In Vitro Proarrhythmia Assay (CiPA), to assess the clinical cardiac safety of compounds prior to Phase I clinical trials.^17^ One essential requirement of the CiPA is to assess the electrophysiological effects of new drugs on hIPSC‐CMs assays. In 2016, Burridge et al established the DOX‐induced cardiomyocyte injury model based on hiPSC‐CMs.^18^ However, they found that dexrazoxane (DEX), a recognized cardioprotective drug used for treating DOX‐induced cardiotoxicity, whose properties were confirmed by animal experiments and clinical trials, could not alleviate the toxicity caused by DOX on hiPSC‐CMs cultured for 30 days.^18^ The immaturity of hiPSC‐CMs might be the leading cause of different responses to the same drug in the heart in vivo and in vitro.

To better understand the mechanism of DOX‐mediated cardiotoxicity in cardiomyocytes of different maturation levels, we chose 30‐day‐old (day 30) and 60‐day‐old (day 60) hiPSC‐CMs to represent immature and relatively mature cardiomyocytes, respectively. We suggest that the level of maturity may affect the modelling of DOX‐induced cardiotoxicity.

## 2 MATERIALS AND METHODS

### 2.1 The hiPSC culture

HiPSCs were purchased from the Cellapy Biological Technology Company. HiPSCs (Cellapy) were routinely maintained in PSCeasy culture medium (Cellapy) and passaged every 3‐4 days using 0.5 mmol/L EDTA (Ethylene Diamine Tetraacetic Acid, Cellapy). Cell lines were used between passages 20 and 85. All cultures were routinely tested for mycoplasma using a MycoAlert Plus Kit (Lonza).

### 2.2 Cardiac differentiation of hiPSCs and purification

HiPSCs were split at 1:8 to 1:12 ratios using EDTA, and grown for 3‐4 days until they reached ~85% confluence. The media were changed to CDM3,^19^ which consisted of RPMI 1640 (Corning), 500 µg/mL Oryza sativa‐derived recombinant human albumin (Oryzogen Sciencell), and 213 µg/mL L‐ascorbic acid 2‐phosphate (Sigma‐Aldrich). The media were changed every 48 hours. On day 0, the media were changed to CDM3 supplemented with 6 µmol/L CHIR99021 (MedChem Express).^20^, ^21^ On day 2, the media were changed to CDM3 supplemented with 2 µmol/L Wnt–C59 (Biorbyt). The media were changed on day 4 and every other day for CDM3. Contracting cells were observed from day 8. On day 10, the media were changed to a purification medium made using RPMI 1640 (no glucose) (Corning), 500 µg/mL recombinant human albumin, and 213 µg/mL L‐ascorbic acid 2‐phosphate. The medium was replaced with RPMI 1640 (Corning) supplemented with 500 µg/mL recombinant human albumin 48 hours before the experiment in order to avoid antioxidant effects.

### 2.3 Flow cytometry cardiac differentiation efficiency

For assessment of cardiac differentiation efficiency, cells on day 15 of differentiation were dissociated using Cell Dissociation Solution (Cellapy) for 25 minutes at 37°C and transferred to flow cytometry tubes (BD Biosciences). Cells were then fixed with 4% paraformaldehyde (PFA) for 10 minutes, permeabilized with 0.1% saponin for 20 minutes, and stained using 1:100 mouse monoclonal IgG1 TNNT2 (Santa Cruz) for 30 minutes at RT. Isotype control Alexa Fluor 594 mouse IgG (Life Technology) was used to establish gating. Cells were then analysed using a flow cytometre.

### 2.4 Cardiomyocyte plating and drug treatment

Cells were separately cultured to 25 and 55 days and then immediately used for experiments. The hiPSC‐CMs were dissociated using Cell Dissociation Solution (Cellapy) for 25 minutes at 37°C, centrifuged at 1000 rpm for 2 minutes and plated onto coverslips, 12‐well cell culture plates or 96‐well culture plates coated with matrigel (Corning) 5 days before experimentation. Doxorubicin hydrochloride (Sigma‐Aldrich) was resuspended to stock solution 1000 μmol/L in PBS. For treatments on hiPSC‐CMs, 2.5 µmol/L DOX was diluted in P11a and cells were treated for 24 hours. For DEX treatment, hiPSC‐CMs were co–treated with 100 μmol/L of DEX (Selleck) with DOX. For N‐Acetyl‐L‐Cysteine (NAC) treatment, hiPSC‐CMs were co‐treated with 1 mmol/L NAC (Sigma‐Aldrich) with DOX.

### 2.5 Quantitative real‐time PCR

Total mRNA was isolated using TRIzol, and 1 µg of the mRNA was used to synthesize cDNA using the GoScript Reverse Transcription System (Promega). A concentration of 0.25 µL of the reaction mixture was used to quantify gene expression by qPCR using SYBR^®^ Premix Ex Taq (TaKaRa). Quantitative real‐time (RT) PCR conditions were as follows: initial denaturation at 94°C for 4 minutes followed by 40 cycles at 94°C for 1 minute, annealing for 1 minute at 56 and 72°C for 1 minute, with a final extension at 72°C for 10 minutes. Expression values were normalized to the average expression of GAPD. Primer sequences are shown in Table S1.

### 2.6 Immunofluorescence staining

The cells were plated on 20 mm coverslips and were fixed with 4% PFA for 20 minutes. Then, after washing with PBS three times for 10 minutes, the cells were successively treated with 0.2% Triton X‐100 (Sigma‐Aldrich) for 30 minutes, washed as above, and treated with 3% bovine serum albumin (BSA, Solarbio) at room temperature. Primary antibodies included OCT4 (1:100, Abcam), SSEA‐4 (1:100, Santa Cruz), NANOG (1:100, Abcam), TRA‐1‐60 (1:100, Santa Cruz), TNNT2 (1:100, Santa Cruz), α‐actinin (1:100, abcam), CX43 (1:100, Santa Cruz) and γ‐H2A.X (1:200, Abcam). Cells were washed and then incubated for 45 minutes at RT in the dark with 1:200 Alexa Fluor secondary antibodies (Life Technology). Cells were washed again as above, mounted with Fluoroshield Mounting Medium with DAPI (4, 6‐diamino‐2‐phenylindole), and imaged using a Confocal Microscope (Carl Zeiss, LSM 510 Meta).

### 2.7 T‐tubules fluorescent staining

After preparing the 100 μmol/L di‐8‐ANEPPS in 20%(w/v) Pluronic‐F127 (Invitrogen) in DMSO from a stock solution of 2 mmol/L di‐8‐ANEPPS in DMSO, cells were incubated with lipophilic fluorescent indicator Di‐8‐ANEPPS (10 μmol/L, Invitrogen) prepared in the cell culture medium for 15 minutes at 37°C, and then washed thrice using PBS. Cells were then fixed with 4% PFA for 20 minutes at RT, washed again, mounted with Fluoroshield Mounting Medium with DAPI and imaged using the confocal microscope.

### 2.8 Calcium (CA2+) imaging

The hiPSC‐CMs were dissociated and seeded in 24mm × 24mm coverslips for calcium imaging. Cells were loaded with 5 μmol/L Fura‐2, AM (Invitrogen) and 0.02% Pluronic F‐127 (Invitrogen) in Tyrodes solution (137 mmol/L NaCl, 5.4 mmol/L KCl, 1 mmol/L MgCl2, 10 mmol/L glucose, 1.8 mmol/L CaCl2, 1mM NaH2PO4 and 20 mmol/L HEPES at pH 7.4 with NaOH at 25°C) for 10 minutes at 37°C. Following Fura‐2, AM loading, cells were washed three times with Tyrodes solution. Ca2+ transients imaging was collected using the confocal microscope with a 40x lens using Zen software (Carl Zeiss). Cells were perfused with calcium‐free Tyrodes solution containing 0.5 mmol/L EGTA, and then the 5 mmol/L caffeine prepared in the same calcium‐free Tyrodes solution was delivered via perfusion apparatus. The analysis was performed using Zeiss physiology software.

### 2.9 Cell viability assay

The hiPSC‐CMs were passaged and cultured in 96‐well plates at 8×104 cells/well. After DOX treatment for 24 hours, 10 µL of CCK–8 (Cell Counting Kit‐8, Dojindo) was added directly to each well in the 96‐well plates, which were then incubated at 37°C for 3 hours; absorbance was read at 450 nm.

### 2.10 TUNEL staining

Cells were stimulated with DOX of different concentrations for 36 hours. Apoptosis was measured using a TUNEL assay kit (Promega). The cells were plated on 20 mm coverslips, fixed with 4% PFA for 20 minutes at RT and washed twice for 5 minutes with PBS. Next, cells were treated with 0.2% Triton X‐100 for 30 minutes and washed twice for 5 minutes with PBS. Then the cells were pre‐incubated with terminal deoxynucleotidyl transferase buffer for 10 minutes at room temperature. In the absence of light, the reaction buffer was added to the cells, and the coverslips were incubated in a humid atmosphere at 37°C for 1 hour. Coverslips were washed again three times for 5 minutes, mounted with Fluoroshield Mounting Medium with DAPI, imaged using the confocal microscope and analysed using Imagej software.

### 2.11 Measurement of mitochondrial transmembrane potential (Δψm) loss

Mitochondrial depolarization was monitored with the potentiometric dye JC‐1 using the Mitoprobe assay kit (Invitrogen) in accordance with the manufacturer's instructions. JC‐1 accumulates in polarized mitochondria with a resting membrane potential and fluoresces red. However, during ΔΨm loss, JC‐1 aggregates are released from the mitochondria, which results in a green fluorescence. Thus, in order to assess mitochondrial depolarization, treated cells were stained with 2 μmol/L JC‐1 for 20 minutes at 37°C at 5% CO2, washed and resuspended in fresh media; and then red and green fluorescence were monitored using High Content Analysis (MetaXpress).

### 2.12 Mitochondrial reactive oxygen species assay

In order to determine the levels of mitochondrial superoxide using fluorescence microscopy, the cells were grown in a 20 mm glass slide. Following treatment, MitoSOX (Invitrogen) reagent was dissolved in dimethyl sulfoxide (5 mmol/L), diluted to 5 µmol/L in serum‐free medium, and was then added to the cells; this was followed by incubation for 10 minutes at 37°C; the cells were then washed twice with PBS. Subsequently, the cells were fixed with 4% PFA for 20 minutes at RT, washed again, mounted with Fluoroshield Mounting Medium with DAPI, imaged using the confocal Microscope and analysed using High Content Analysis.

### 2.13 Intracellular reactive oxygen species assay

The intracellular reactive oxygen species (ROS) level was determined using 2', 7'‐dichlorodihydrofluorescein diacetate (DCFH‐DA) (Beyotime). The cells were grown in the 20 mm glass slide. Following treatment, DCFH‐DA reagent was diluted to 10 µmol/L in serum‐free medium and was then added to the cells; this was followed by incubation for 20 minutes at 37°C; the cells were then washed twice with PBS. Subsequently, the cells were fixed with 4% PFA for 20 minutes at RT, washed again, mounted with Fluoroshield Mounting Medium with DAPI, imaged using the confocal microscope and analysed using High Content Analysis.

### 2.14 Mitochondria staining

In order to determine the distribution and level of mitochondria, the cells were grown in the 20 mm glass slide. Mitotracker (Invitrogen) reagent was diluted to 1 µmol/L in serum‐free medium, and was then added to the cells; this was followed by incubation for 30 minutes at 37°C; the cells were then washed twice with PBS. Subsequently, the cells were fixed with 4% PFA for 20 minutes at RT, washed again, mounted with Fluoroshield Mounting Medium with DAPI, imaged using the confocal microscope and analysed using High Content Analysis.

### 2.15 Statistical methods

Data were analysed using the SPSS Statistics 20 (IBM) package and graphed using Prism (GraphPad). The data are presented as (mean ± SEM). Comparisons were conducted using the one‐way ANOVA test, followed by either the All Pairwise Multiple Comparison Procedures (Sidak) method or an unpaired, two–tailed Student's *t *test.

## 3 RESULTS

### 3.1 Generation of hiPSC‐CMs

**Figure 1:**
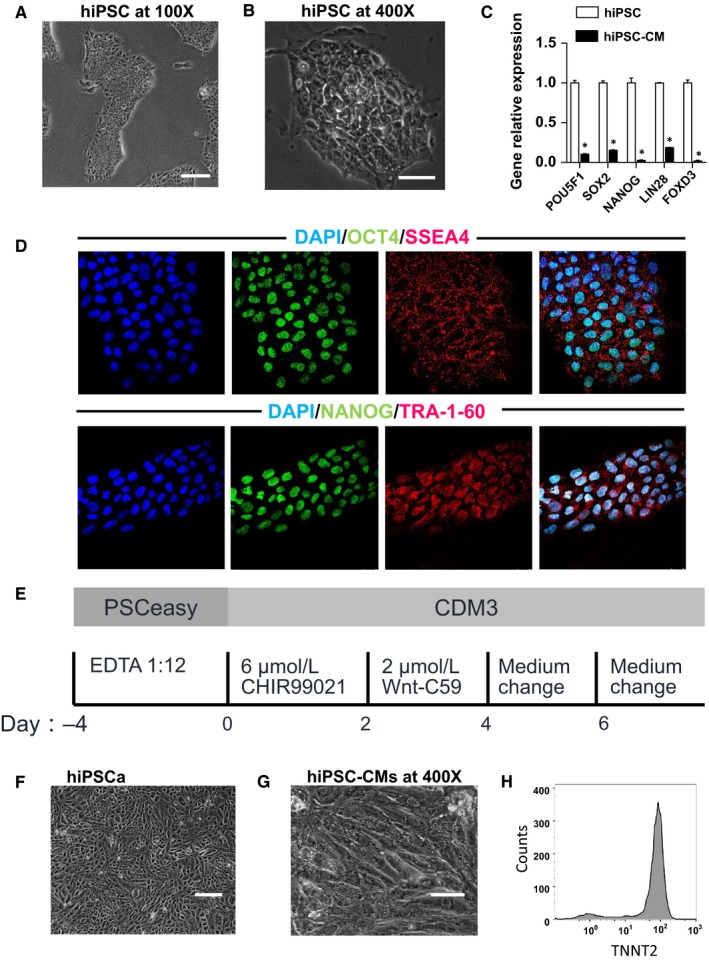
Cardiomyocytes are differentiated from hiPSC. (A), hiPSCs observed under low power microscopy (scale bar = 200 μm). (B), hiPSCs observed under high power microscopy (scale bar = 50 μm). (C), The multipotency related genes expression of the hiPSC and human‐induced pluripotent stem cell‐derived cardiomyocytes (hiPSC‐CMs) by QPCR assay (n = 3). (D), Representative immunofluorescence staining of hiPSC for markers of stem cell, including OCT4, SSEA4, NANOG and TRA‐1‐60 (scale bar = 50 μm). (E), The protocol of hiPSCs differentiating into hiPSC‐CMs. (F), hiPSC‐CMs observed under low power microscopy (scale bar = 200 μm). (G), hiPSC‐CMs observed under high power microscopy (scale bar = 50 μm). (H), Purified hiPSC‐CMs expressing the cardiac marker TNNT2 by flow cytometry analysis. *P < 0.05 doxorubicin vs control. Error bars represent SEM

In an effort to ensure reproducibility, we cultured hiPSCs under chemically defined conditions, and hiPSCs showed normal colony morphology (Figure 1A,B). Then we examined the pluripotency of hiPSCs using immunofluorescence staining for pluripotent markers OCT4, SSEA‐4, NANOG and TRA‐1‐60 (Figure 1D). Using a small molecule‐based directed differentiation method (Figure 1E), we were able to generate high purity CMs from the hiPSCs cultured as a monolayer. The expression of multipotency related genes *POU5F1, SOX2, NANOG, LIN28 and FOXD3* decreased significantly after differentiating into hiPSC‐CMs (Figure 1C). Approximately 8 days after induction, the differentiated cells started to contract spontaneously (Figure 1F,G). Flow cytometry analysis indicated that the hiPSC‐CMs were highly pure cellular populations, with more than 95% of cells expressing the cardiac marker TNNT2 (Figure 1H).

### 3.2 hiPSC‐CMs matured over time when cultured in vitro

**Figure 2:**
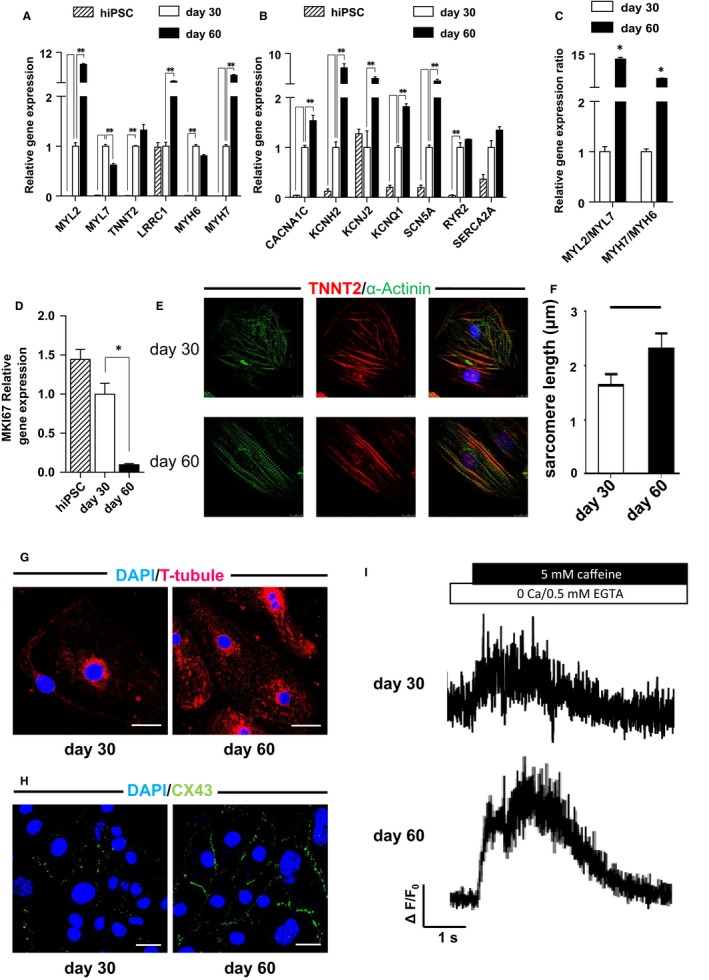
Different characteristics of human‐induced pluripotent stem cell‐derived cardiomyocytes cultured 30 d and 60 d, respectively. (A), Structure genes expression quantities of the two groups by QPCR assay (n = 3). (B), Functional genes expression quantities of the two groups by QPCR assay (n = 3). (C), The expression ratio of ventricular‐like structure genes and atrial‐like genes in the day 30 and day 60 hiPSC‐CM groups (n = 3). (D), Proliferation‐related gene MKI67 expression quantity of the two groups by QPCR assay (n = 3). (E), Immunofluorescent staining for TNNT2 and phalloidin to demonstrate sarcomeric organization in the two hiPSC‐CM groups (scale bar = 25 µm). (F), Quantified sarcomere length of day 30 and day 60 CMs. (G), Immunofluorescent staining for T‐tubules in the two groups (scale bar = 25 µm). (H), Immunofluorescent staining for connexin 43 in the two groups (scale bar = 50 µm). (I), Representative Ca2+ transient traces from the two groups followed by caffeine exposure. *P < 0.05 doxorubicin vs control. Error bars represent SEM

We found that the maturity of hiPSC‐CMs increased with culture time, so we chose hiPSC‐CMs that were 30 days old and 60 days old; we compared their characteristics in terms of molecular expression, myofilament structure, gap junctions and calcium transients. We first compared the changes in the gene expression profiles of structural and functional genes using quantitative real‐time PCR. The expression of sarcomeric genes (such as *MYL2, LRRC1* and *MYH7*) and ion channel genes (such as *CACNA1C, KCNH2, KCNJ2, KCNQ1* and *SCN5A*) were significantly up‐regulated in the day 60 hiPSC‐CMs compared to the day 30 hiPSC‐CMs (Figure 2A,B). Meanwhile, the *MYH7/MYH6* and *MYL2/MYL7* gene expression ratios increased over time (Figure 2C), implying an increased percentage of ventricular‐like cells in the day 60 hiPSC‐CMs.^22^ The number of differentiated cells had increased in the day 30 hiPSC‐CMs, whereas multiplication of cells in the day 60 hiPSC‐CMs had been inhibited. We also observed that the cell cycle‐related genes *MKI67* (encoding the proliferation marker protein KI67) were richly expressed in day 30 hiPSC‐CMs (Figure 2D), signifying immaturity. Immunofluorescence staining showed that day 60 hiPSC‐CMs possessed distinct, well‐developed and abundant sarcomeres that were organized in parallel, in contrast with the day 30 hiPSC‐CMs (Figure 2E). The sarcomere length of day 60 CMs is longer than day 30 cardiomyocytes as shown in Figure 2F (2.308 ± 0.091 41, n = 10 vs 1.638 ± 0.059 61, n = 12). Electron microscopy images indicated that immature mitochondria were distributed surrounding the nucleus, and the sarcomere was underdeveloped in day 30 CMs (Figure S1A). Whereas mature mitochondria were distributed along the myofilament of day 60 CMs (Figure S1B). The day 60 hiPSC‐CMs also had defined T‐tubules, which are invaginations of the cardiomyocyte plasma membrane along the boundary between adjacent sarcomeres, and which are responsible for transmitting the action potential from the sarcolemma to the sarcoplasmic reticulum (SR)^23^ (Figure 2G). In addition, connexin‐43 (CX43), the predominant cardiac gap junction protein, was observed to be more abundantly distributed throughout the cardiomyocyte membrane in the day 60 hiPSC‐CMs than in the day 30 cells (Figure 2H). Calcium transients comprise one of the characteristics of cardiomyocyte: we assessed calcium transients by loading single cells with Fura‐2, AM in order to probe the differential characteristics of the two group cells in terms of calcium transients. The day 30 cells showed limited capacity to release Ca2+ when treated with caffeine (SR Ca2+ channel opener) compared with the day 60 cells (Figure 2I), confirming that immature hiPSC‐CMs had significant lower level of SR function (0.8167 ± 0.026 03, n = 3 vs 1.307 ± 0.020 28, n = 3; day 30 vs day 60, *P* < 0.01). After measuring the spontaneous calcium transient, we found that the amplitude of day 60 CMs was higher than day 30 CMs (15.84 ± 0.4439, n = 5 vs 10.70 ± 0.250 9, n = 5), and the transient duration of day 60 CMs was also longer (1.276 ± 0.022 84, n = 3 vs 0.701 7 ± 0.011 26, n = 3) (Figure S2A,B). These results suggested that the day 60 hiPSC‐CMs were more mature in terms of structural and functional phenotypes than the day 30 hiPSC‐CMs.

### 3.3 Maturation decreased the cytotoxicity of DOX in hiPSC‐CMs

**Figure 3:**
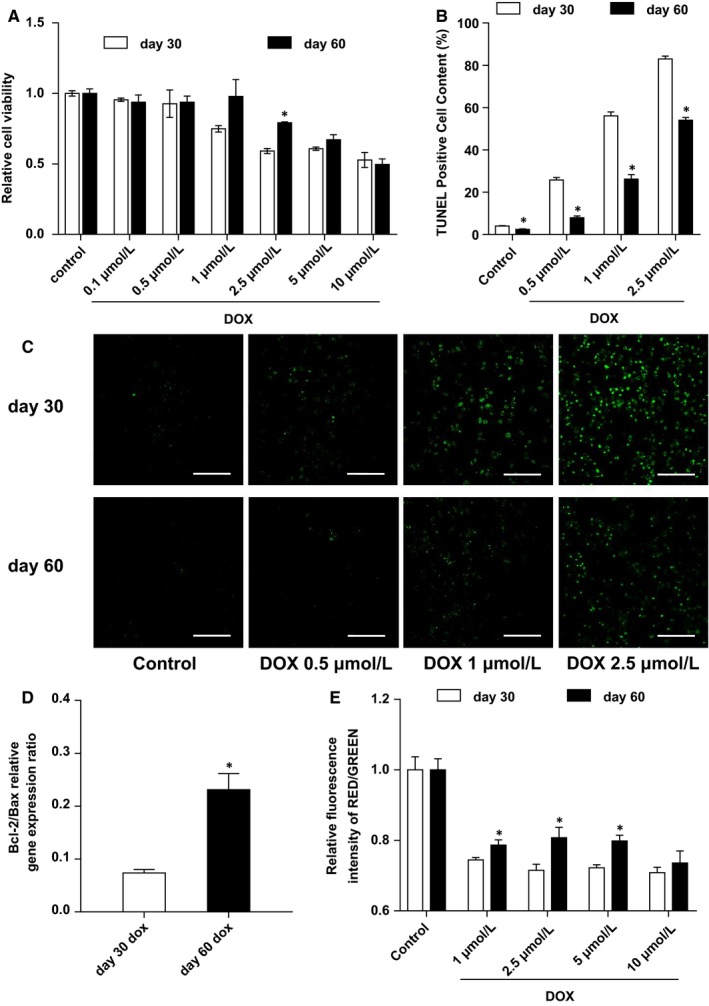
Day 30 human‐induced pluripotent stem cell‐derived cardiomyocytes (hiPSC‐CMs) are more vulnerable to Doxorubicin (DOX) compared with day 60 hiPSC‐CMs. (A), Detection of relative cell viability in the two groups after 24 h treatment with DOX of different concentrations by CCK8 assay (n = 3). (B), Quantitative analysis of TUNEL staining of the two groups treated with DOX of different concentrations (n = 3). (C), Cell apoptosis assay in the two groups after 24 h treatment with DOX of different concentrations demonstrated by TUNEL staining. (D), Relative quantitative analysis of BCL‐2/BAX expression ratio of the two groups after 24 h treatment with DOX (n = 3). (E), Mitochondrial membrane potential assay of the two groups after 24 h treatment with DOX of different concentrations by JC‐1 probe (n = 3). *P < 0.05 DOX vs control. Error bars represent SEM

We have suggested that the degree of maturity influences the cells’ response to DOX and affects the modelling of DOX‐induced cardiotoxicity. Therefore, we measured the cell viability of cells exposed to DOX. Notably, the day 30 hiPSC‐CMs showed significantly reduced cell viability compared to the day 60 group at concentrations of 2.5 μmol/L DOX (Figure 3A). To reveal the cause of viability reduction, we then assessed cellular apoptosis using TUNEL staining. Compared to the day 60 cells, the day 30 hiPSC‐CMs treated with DOX displayed a significantly increased apoptosis rate (Figure 3B,C). Relative quantification of Bcl‐2/Bax expression ratios of the two hiPSC‐CM groups after 24 hours of treatment with 2.5 μmol/L DOX showed a higher apoptosis level in the day 30 hiPSC‐CMs (Figure 3D). The mitochondrial membrane potential assay by JC‐1 probe showed a more obvious decrease in the day 30 cells (Figure 3E). These results demonstrated that the day 30 hiPSC‐CMs are more sensitive to the cytotoxicity of DOX than the day 60 hiPSC‐CMs.

### 3.4 Maturation led to more ROS production in hiPSC‐CMs

**Figure 4:**
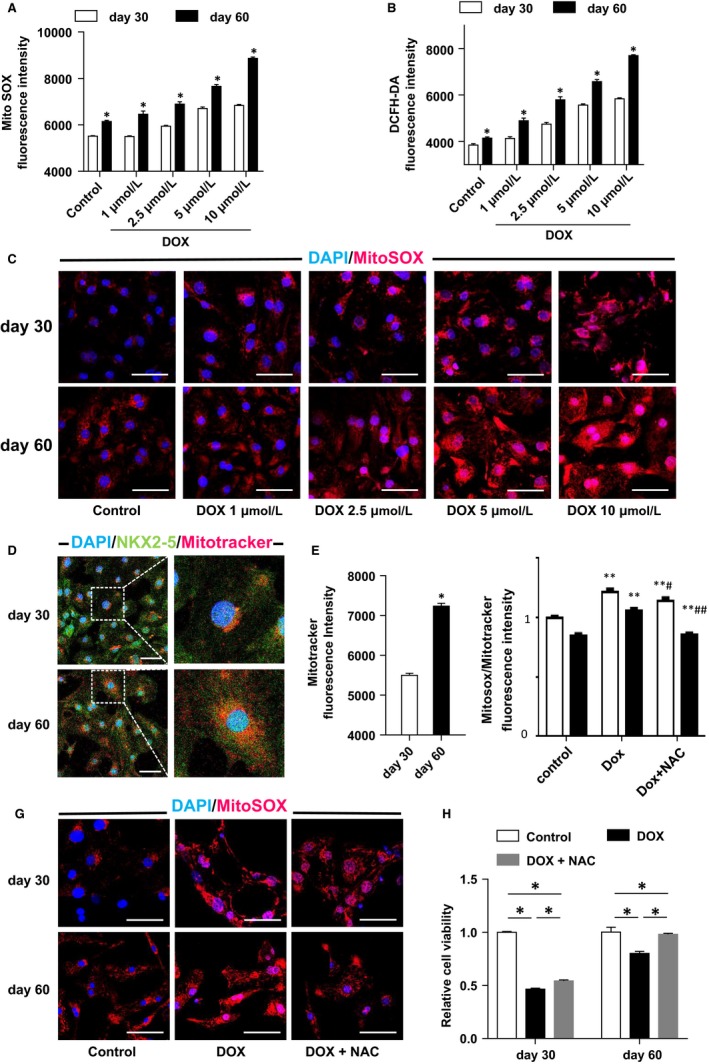
Higher reactive oxygen species (ROS) is produced in the day 60 human‐induced pluripotent stem cell‐derived cardiomyocytes (hiPSC‐CMs) compared with day 30 hiPSC‐CMs. (A), Mitochondrial ROS production under treatment by Doxorubicin (DOX) of different concentrations demonstrated by MitoSOX immunofluorescent staining (n = 3). (B), The baseline level of MitoSOX fluorescence intensity of the two groups (n = 3). (C), The fluorescence intensity of DCFH‐DA, representing intracellular ROS production (n = 3). (D), Cells incubated with DOX for 24 h and mitochondrial ROS production was detected using MitoSOX fluorescent staining (scale bar = 50 µm). (E), Immunofluorescent staining for mitochondria in the two groups by Mitotracker fluorescent probe (scale bar = 50 µm). (F), Quantitative analysis of Mitotracker fluorescence intensity (n = 3). (G), Effects of DOX to ROS production with or without N–acetyl–L–cysteine (NAC) demonstrated by MitoSOX quantitative analysis (n = 3). (H), Effects of DOX to ROS production with or without NAC demonstrated by MitoSOX immunofluorescent staining (scale bar = 50 µm). (I), Effects of DOX to the cell viability with or without NAC treatment demonstrated by CCK8 assay (n = 3). *P < 0.05 DOX vs control, ^#^ *P* < 0.05 DOX + NAC vs DOX. Error bars represent SEM

Doxorubicin‐induced cardiotoxicity is known to have two major mechanisms: induction of ROS through redox cycling causing injury to membrane structure, and binding to Top2 causing DNA damage.^24^, ^25^, ^26^, ^27^ We assessed the production of mitochondrial ROS after DOX treatment and found that DOX treatment led to an increase in mitochondrial ROS levels in a dose‐dependent manner in the hiPSC‐CMs (Figure 4A,C). The intracellular ROS production exhibited the same trend (Figure 4B). To explore the underlying mechanisms, we assessed the mitochondrial mass and distribution of both cell groups, and found that the day 60 hiPSC‐CMs possessed more abundant mitochondria distributed around the cells, whereas the mitochondria of the day 30 hiPSC‐CMs was less abundant and was accumulated around the nucleus in the cytoplasm (Figure 4D,E). Furthermore, in applying NAC, a common antioxidant, we found it could attenuate ROS production in the day 60 hiPSC‐CMs to a greater extent than in the day 30 group (Figure 4F,G). In order to determine the impact of ROS overproduction on cell viability reduction, we analysed the cell viability following co‐treatment with antioxidant NAC. Our results showed that NAC could restore cell viability in both groups, but more evidently so in the day 60 group (Figure 4H).

Consequently, we may conclude that DOX can induce ROS production in hiPSC‐CMs in a dose‐dependent manner and that DOX leads to more ROS production in the day 60 hiPSC‐CMs.

### 3.5 DOX led to more severe DNA damage in less mature hiPSC‐CMs through TOP2α

However, the above results do not explain the phenomenon of more severe toxicity in the day 30 hiPSC‐CMs, so we considered the other pathway of DNA damage. DOX, targeting Top2, plays its cytotoxic role due to the formation of a Top2‐DOX‐DNA complex. Top2 plays an important role in maintaining the DNA topology.^28^ There are two Top2 isozymes in human cells: Top2α and Top2β.^29^ Top2α is only expressed in proliferative cells and tumour cells, and is highly expressed in the G2/M period of cell cycle. DOX is thought to bind to Top2α, which has a highly elevated expression in cancer cells.^30^ By contrast, Top2β is present in all cells. It is worth noting that adult mammalian cardiomyocytes express TOP2β but contain no detectable TOP2α. There is substantial evidence that Top2β is predominantly responsible for DOX‐induced cardiotoxicity via DNA damage.^31^ The role of Top2β was verified by a previous study showing that cardiomyocyte‐specific deletion of Top2β protects mice from the development of DOX‐induced progressive heart failure.^32^

**Figure 5:**
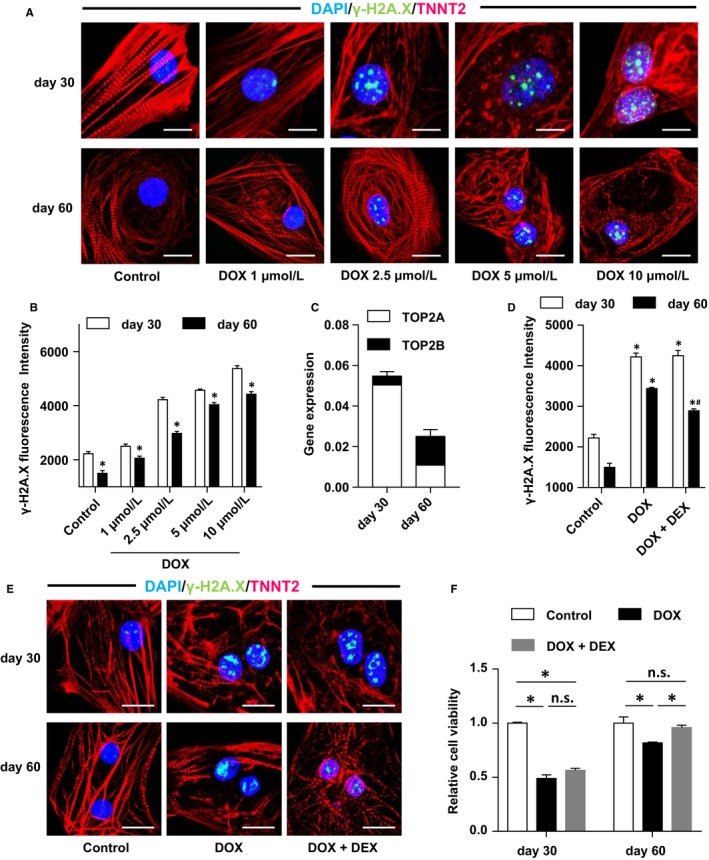
More severe DNA damage by Doxorubicin (DOX) in day 30 human‐induced pluripotent stem cell‐derived cardiomyocytes (hiPSC‐CMs) via TOP2α. (A), Detection of DNA damage using immunofluorescent staining for γ‐H2A.X after 24 h treatment of DOX (scale bar = 15 µm). (B), Quantitative analysis of γ‐H2A.X fluorescence intensity (n = 3). (C), Expression quantities of genes TOP2A and TOP2B in the two groups by QPCR assay (n = 3). (D), Effects of DOX to the DNA damage with or without dexrazoxane (DEX) treatment demonstrated by γ‐H2A.X quantitative analysis (n = 3). (E), Effects of DOX to the DNA damage with or without DEX treatment demonstrated by γ‐H2A.X immunofluorescent staining (scale bar = 15 µm). (F), Effects of DOX to the cell viability with or without DEX treatment demonstrated by CCK8 assay (n = 3). *P < 0.05 DOX vs control, ^#^ *P* < 0.01 DOX + DEX vs DOX. Error bars represent SEM

We assessed the level of double‐stranded DNA damage by detecting phosphorylated H2A histone family member X (γ‐H2A.X). We observed a dose‐dependent increase in DNA damage, which was significantly higher in the day 30 hiPSC‐CMs (Figure 5A,B). Given that the day 30 hiPSC‐CMs were more proliferative than the day 60 cells, it was necessary to explore the gene expression of TOP2α and TOP2β. The test showed that the day 60 hiPSC‐CMs expressed a higher percentage of *TOP2B*, whereas the day 30 hiPSC‐CMs expressed mainly *TOP2A* (Figure 5C). Therefore, we have suggested that the difference in DNA damage caused by DOX between two groups of cells may be related to the expression of different TOP2 subtypes. To verify the hypothesis, we used DEX, which induces Top2β depletion and antagonizes DOX‐induced cardiotoxicity by preventing DOX‐induced DNA damage, whilst not decreasing the anti‐tumour efficacy at the same time.^33^ In our findings, DNA damage signal γ‐H2A.X is more significantly reduced with treatment of DEX in the day 60 cells than in the day 30 cells, which supports our hypothesis (Figure 5D,E). In order to establish the impact of DNA damage to cell viability reduction, we analysed cell viability following co‐treatment with DEX. The results showed that DEX only restored cell viability in the day 60 group (Figure 5F). These results indicated that DEX is capable of reducing DOX‐induced DNA damage in hiPSC‐CMs via depletion of Top2β in the day 60 group, which suggests that the day 60 hiPSC‐CMs may serve as a better disease model for DOX‐induced cardiotoxicity. Moreover, cardiomyocytes of a low degree of maturation are more sensitive to DOX, which may be related to the high content of TOP2α and thus to more severe DNA damage.

## 4 DISCUSSION

**Figure 6:**
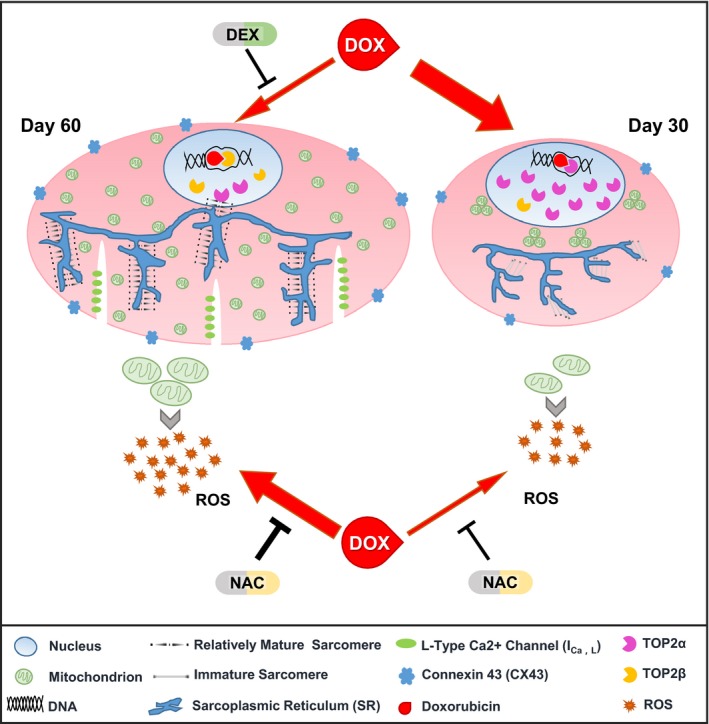
Schematic illustration of doxorubicin (DOX)‐induced cardiotoxicity in human‐induced pluripotent stem cell‐derived cardiomyocytes (hiPSC‐CMs) cultured 30 d and 60 d respectively. The day 60 hiPSC‐CMs were relatively mature, with well‐developed and distinct sarcomeres, defined T‐tubules, and more abundant connexin‐43 and mitochondria, compared to the day 30 hiPSC‐CMs. When treated with Doxorubicin (DOX), the day 60 hiPSC‐CMs produced more reactive oxygen species (ROS) and better simulated the physiological processes of ROS overproduction, although both groups responded to N‐Acetyl‐L‐Cysteine. In addition, the day 30 hiPSC‐CMs mainly expressed TOP2α rather than TOP2β, and failed to mimic DOX‐induced cardiotoxicity in the pathophysiological process of DNA damage

We have shown that DOX injury to hiPSC‐CMs occurs in a dose‐dependent manner in terms of cell viability, oxidative stress level and DNA damage, and that the day 30 hiPSC‐CMs and day 60 hiPSC‐CMs respond differently to DOX. The different responses correlate with the degree of maturity of the two groups. The day 60 hiPSC‐CMs had more mature phenotypes in terms of molecular expression, myofilament structure, gap junctions and calcium transients; the day 30 hiPSC‐CMs by comparison are relatively immature. The overproduction of ROS is one of the most important mechanisms of DOX‐induced cardiotoxicity^24^, ^25^, ^26^, ^27^ and ROS originates mainly from mitochondria. The mitochondria content of the day 30 hiPSC‐CMs is much lower than that of the day 60 hiPSC‐CMs. The day 60 hiPSC‐CMs simulated the physiological processes of ROS overproduction better than the day 30 hiPSC‐CMs. However, these results contradict the observations that day 30 hiPSC‐CMs present much more reduction in cell viability, suggesting that ROS might play a minor role of DOX‐induced cardiotoxicity in less mature hiPSC‐CMs. Moreover, previous research has shown that there are differences in the type and content of *TOP2*, the target of DOX, in cells with or without proliferative capacity.^29^ We found that the day 30 hiPSC‐CMs mainly express *TOP2A*, the gene for TOP2α, similar to tumour cells, whereas day 60 hiPSC‐CMs predominantly express *TOP2B*, the gene for TOP2β, which is similar to the phenotype of adult cardiomyocytes; therefore, day 60 hiPSC‐CMs can more accurately mimic DOX‐induced cardiotoxicity in the pathophysiological process of DNA damage (Figure 6).

The maturity of hiPSC‐CMs increases with culture time,^34^ something that has been repeatedly confirmed. However, this article is perhaps the first to investigate the effects of maturity on disease modelling. In 2016, Paul et al established a DOX‐induced cardiotoxicity model based on hiPSC‐CMs, and showed that the recognized DOX cardiotoxicity cardioprotective drug DEX could not alleviate the toxicity of DOX on hiPSC‐CMs cultured for 30 days.^18^ In this study, we have shown that DEX has a protective effect on day 60 hiPSC‐CMs but no protective effect on day 30 hiPSC‐CMs, the latter which might have been affected by the main expression of TOP2α instead of TOP2β. Given that binding to TOP2β is one of the important mechanisms of DOX‐induced cardiotoxicity, we conclude that day 60 hiPSC‐CMs, the more mature group, serve as a better model of DOX‐induced cardiotoxicity. As illustrated above, we found that the degree of maturity of hiPSC‐CMs has an impact on the model of DOX‐induced cardiotoxicity. On the one hand, relatively mature cardiomyocytes can better model DOX‐induced cardiotoxicity for pathogenesis research and drug screening; on the other hand, the effects of the degree of maturation should be taken into consideration when establishing other disease models based on hiPSC‐CMs. In addition, building a disease model requires hiPSC‐CMs of the same level of maturity in order to avoid inconsistent experimental results. Therefore, the significance of this study is that it sheds light on the importance of optimizing the establishment of the disease model based on hiPSC‐CMs.

Previous studies have confirmed that as the heart of a human or a mouse develops, the expression of *TOP2A* gradually decreases, and the expression of *TOP2B* generally increases.^35^, ^36^, ^37^ We speculate that the sensitivity of age‐related DOX‐induced cardiotoxicity may be related to the different levels of expression of these *TOP2* genes, which might help guide clinical medication and prevent DOX cardiotoxicity.

We aimed to clarify this phenomenon, rather than studying DOX‐induced cardiotoxicity itself, and so we used the hiPSC‐CMs of a healthy individual instead of those taken from patients suffering from DOX‐induced cardiomyopathy. However, it is better to choose hiPSC‐CMs from patients who are suffering from DOX‐induced cardiomyopathy for pathogenesis studies and drug screening. The technology used for hiPSC‐CM analyses has developed rapidly over the past 10 years. HiPSC‐CM analyses take into account the overall genetic background of a specific individual, and thus have many advantages over animal models. However, hiPSC‐CMs do not match the phenotype of adult cardiomyocytes in terms of maturity. There are a number of ways to promote the maturation of hiPSC‐CMs, but these methods only allow hiPSC‐CMs to approach the phenotype of late foetal cardiomyocytes, which is far from the phenotype of adult cardiomyocytes.^23^ This is an emerging technology, and there remains considerable room for improvement.

## CONFLICT OF INTEREST

The authors declare that there is no conflict of interest in relation to the experiments or this paper.

## References

[1] Smith LA , Cornelius VR , Plummer CJ , et al. Cardiotoxicity of anthracycline agents for the treatment of cancer: systematic review and meta‐analysis of randomised controlled trials*.* BMC Cancer. 2010;10:337. 20587042 10.1186/1471-2407-10-337 PMC2907344

[2] Giordano SH , Lin Y‐L , Kuo YF , Hortobagyi GN , Goodwin JS . Decline in the use of anthracyclines for breast cancer. J Clin Oncol. 2012;30(18):2232‐2239. 22614988 10.1200/JCO.2011.40.1273 PMC3397719

[3] Nabhan C , Byrtek M , Rai A , et al. Disease characteristics, treatment patterns, prognosis, outcomes and lymphoma‐related mortality in elderly follicular lymphoma in the United States. Br J Haematol. 2015;170(1):85‐95. 25851937 10.1111/bjh.13399 PMC5076864

[4] Chihara D , Westin JR , Oki Y , et al. Management strategies and outcomes for very elderly patients with diffuse large B‐cell lymphoma. Cancer. 2016;122(20):3145‐3151. 27351173 10.1002/cncr.30173

[5] Swain SM , Whaley FS , Ewer MS . Congestive heart failure in patients treated with doxorubicin ‐ A retrospective analysis of three trials. Cancer. 2003;97(11):2869‐2879. 12767102 10.1002/cncr.11407

[6] Hequet O , Le QH , Moullet I , et al. Subclinical late cardiomyopathy after doxorubicin therapy for lymphoma in adults. J Clin Oncol. 2004;22(10):1864‐1871. 15143078 10.1200/JCO.2004.06.033

[7] McGowan JV , Chung R , Maulik A , Piotrowska I , Walker JM , Yellon DM . Anthracycline chemotherapy and cardiotoxicity. Cardiovasc Drugs Ther. 2017;31(1):63‐75. 28185035 10.1007/s10557-016-6711-0 PMC5346598

[8] Krischke M , Hempel G , Völler S , et al. Pharmacokinetic and pharmacodynamic study of doxorubicin in children with cancer: results of a "European Pediatric Oncology Off‐patents Medicines Consortium" trial. Cancer Chemother Pharmacol. 2016;78(6):1175‐1184. 27770238 10.1007/s00280-016-3174-8 PMC5114325

[9] Lipshultz SE , Colan SD , Gelber RD , Perez‐Atayde AR , Sallan SE , Sanders SP . Late cardiac effects of doxorubicin therapy for acute lymphoblastic leukemia in childhood. N Engl J Med. 1991;324(12):808‐815. 1997853 10.1056/NEJM199103213241205

[10] Lipshultz SE , Lipsitz SR , Mone SM , et al. Female sex and higher drug dose as risk‐factors for late cardiotoxic effects of doxorubicin therapy for childhood‐cancer. N Engl J Med. 1995;332(26):1738‐1743. 7760889 10.1056/NEJM199506293322602

[11] Pratt CB , Ransom JL , Evans WE . Age‐related adriamycin cardiotoxicity in children. Cancer Treat Rep. 1978;62(9):1381‐1385. 356987

[12] Von Hoff DD , Rozencweig M , Layard M , Slavik M , Muggia FM . Daunomycin‐induced cardiotoxicity in children and adults. A review of 110 cases. Am J Med. 1977;62(2):200‐208. 835599 10.1016/0002-9343(77)90315-1

[13] Potts MB , Vaughn AE , McDonough H , Patterson C , Deshmukh M . Reduced Apaf‐1 levels in cardiomyocytes engage strict regulation of apoptosis by endogenous XIAP. J Cell Biol. 2005;171(6):925‐930. 16344307 10.1083/jcb.200504082 PMC2171313

[14] Konorev EA , Vanamala S , Kalyanaraman B . Differences in doxorubicin‐induced apoptotic signaling in adult and immature cardiomyocytes. Free Radic Biol Med. 2008;45(12):1723‐1728. 18926904 10.1016/j.freeradbiomed.2008.09.006 PMC3039518

[15] Shi J , Zhang L , Zhang YW , Surma M , Mark Payne R , Wei L . Downregulation of doxorubicin‐induced myocardial apoptosis accompanies postnatal heart maturation. Am J Physiol Heart Circ Physiol. 2012;302(8):H1603‐H1613. 22328080 10.1152/ajpheart.00844.2011 PMC3330803

[16] Matsa E , Burridge PW , Wu JC . Human stem cells for modeling heart disease and for drug discovery. Sci Transl Med. 2014;6(239):239ps236. 10.1126/scitranslmed.3008921 PMC4215696 24898747

[17] Sager PT , Gintant G , Turner JR , Pettit S , Stockbridge N . Rechanneling the cardiac proarrhythmia safety paradigm: a meeting report from the Cardiac Safety Research Consortium. Am Heart J. 2014;167(3):292‐300. 24576511 10.1016/j.ahj.2013.11.004

[18] Burridge PW , Li YF , Matsa E , et al. Human induced pluripotent stem cell-derived cardiomyocytes recapitulate the predilection of breast cancer patients to doxorubicin-induced cardiotoxicity. Nat Med. 2016;22(5):547-556. 27089514 10.1038/nm.4087 PMC5086256

[19] Piccini I , Rao J , Seebohm G , Greber B . Human pluripotent stem cell‐derived cardiomyocytes: genome‐wide expression profiling of long‐term in vitro maturation in comparison to human heart tissue. Genomics Data. 2015;4:69‐72. 26484180 10.1016/j.gdata.2015.03.008 PMC4535944

[20] Scuderi GJ , Butcher J . Naturally engineered maturation of cardiomyocytes. Front Cell Dev Biol. 2017;5:50. 28529939 10.3389/fcell.2017.00050 PMC5418234

[21] Davies K , Doroshow JH . Redox cycling of anthracyclines by cardiac mitochondria. 1. anthracycline radical formation by nadh dehydrogenase. J Biol Chem. 1986;261(7):3060‐3067. 3456345

[22] Berthiaume JM , Wallace KB . Adriamycin‐induced oxidative mitochondrial cardiotoxicity. Cell Biol Toxicol. 2007;23(1):15‐25. 17009097 10.1007/s10565-006-0140-y

[23] Hahn VS , Lenihan DJ , Ky B . Cancer therapy‐induced cardiotoxicity: basic mechanisms and potential cardioprotective therapies. J Am Heart Assoc. 2014;3(2):e000665. 24755151 10.1161/JAHA.113.000665 PMC4187516

[24] Ichikawa Y , Ghanefar M , Bayeva M , et al. Cardiotoxicity of doxorubicin is mediated through mitochondrial iron accumulation. J Clin Invest. 2014;124(2):617‐630. 24382354 10.1172/JCI72931 PMC3904631

[25] Champoux JJ . DNA topoisomerases: structure, function, and mechanism. Annu Rev Biochem. 2001;70:369‐413. 11395412 10.1146/annurev.biochem.70.1.369

[26] Wang JC . Cellular roles of DNA topoisomerases: a molecular perspective. Nat Rev Mol Cell Biol. 2002;3(6):430‐440. 12042765 10.1038/nrm831

[27] Carpenter AJ , Porter A . Construction, characterization, and complementation of a conditional‐lethal DNA topoisomerase II alpha mutant human cell line. Mol Biol Cell. 2004;15(12):5700‐5711. 15456904 10.1091/mbc.E04-08-0732 PMC532048

[28] Tewey K , Rowe T , Yang L , Halligan B , Liu L . Adriamycin‐induced DNA damage mediated by mammalian DNA topoisomerase‐II. Science. 1984;226(4673):466‐468. 6093249 10.1126/science.6093249

[29] Zhang S , Liu X , Bawa‐Khalfe T , et al. Identification of the molecular basis of doxorubicin‐induced cardiotoxicity. Nat Med. 2012;18(11):1639‐1642. 23104132 10.1038/nm.2919

[30] Hensley ML , Hagerty KL , Kewalramani T , et al. American society of clinical oncology 2008 clinical practice guideline update: use of chemotherapy and radiation therapy protectants. J Clin Oncol. 2009;27(1):127‐145. 19018081 10.1200/JCO.2008.17.2627

[31] Kamakura T , Makiyama T , Sasaki K , et al. Ultrastructural maturation of human‐induced pluripotent stem cell‐derived cardiomyocytes in a long‐term culture. Circ J. 2013;77(5):1307‐1314. 23400258 10.1253/circj.cj-12-0987

[32] Capranico G , Tinelli S , Austin CA , Fisher ML , Zunino F . Different patterns of gene‐expression of topoisomerase‐II isoforms in differentiated tissues during murine development. Biochem Biophys Acta. 1992;1132(1):43‐48. 1380833 10.1016/0167-4781(92)90050-a

[33] NCBIGene ., Tissue‐specific circular RNA induction during human fetal development of TOP2B, in [EB/OL]. 2018–2‐4, https://www.ncbi.nlm.nih.gov/gene/7155/?report=expression&bioproject=PRJNA270632. Accessed March 5, 2018.

[34] NCBIGene ., Tissue‐specific circular RNA induction during human fetal development of TOP2A, in [EB/OL]. 2018–2‐20, https://www.ncbi.nlm.nih.gov/gene/7153/?report=expression&bioproject=PRJNA270632. Accessed March 5, 2018.

[35] Kremer L , van der Pal H , Offringa M , van Dalen EC , Voute PA . Frequency and risk factors of subclinical cardiotoxicity after anthracycline therapy in children: a systematic review. Ann Oncol. 2002;13(6):819‐829. 12123328 10.1093/annonc/mdf167

[36] Burridge PW , Matsa E , Shukla P , et al. Chemically defined generation of human cardiomyocytes. Nat Methods. 2014;11(8):855‐860. 24930130 10.1038/nmeth.2999 PMC4169698

[37] Burridge PW , Holmstrom A , Wu JC . Chemically defined culture and cardiomyocyte differentiation of human pluripotent stem cells. Curr Protoc Hum Genet. 2015;87:21.3.1‐21.3.15. 10.1002/0471142905.hg2103s87 PMC4597313 26439715

