# Supplementary figures and images for: Doxorubicin‐induced cardiotoxicity is maturation dependent due to the shift from topoisomerase IIα to IIβ in human stem cell derived cardiomyocytes
Source: J Cell Mol Med. 2019 May 20;23(7):4627–39. doi: 10.1111/jcmm.14346 (PMC6584544; doi:10.1111/jcmm.14346)

# Supplemental Figure 1

A

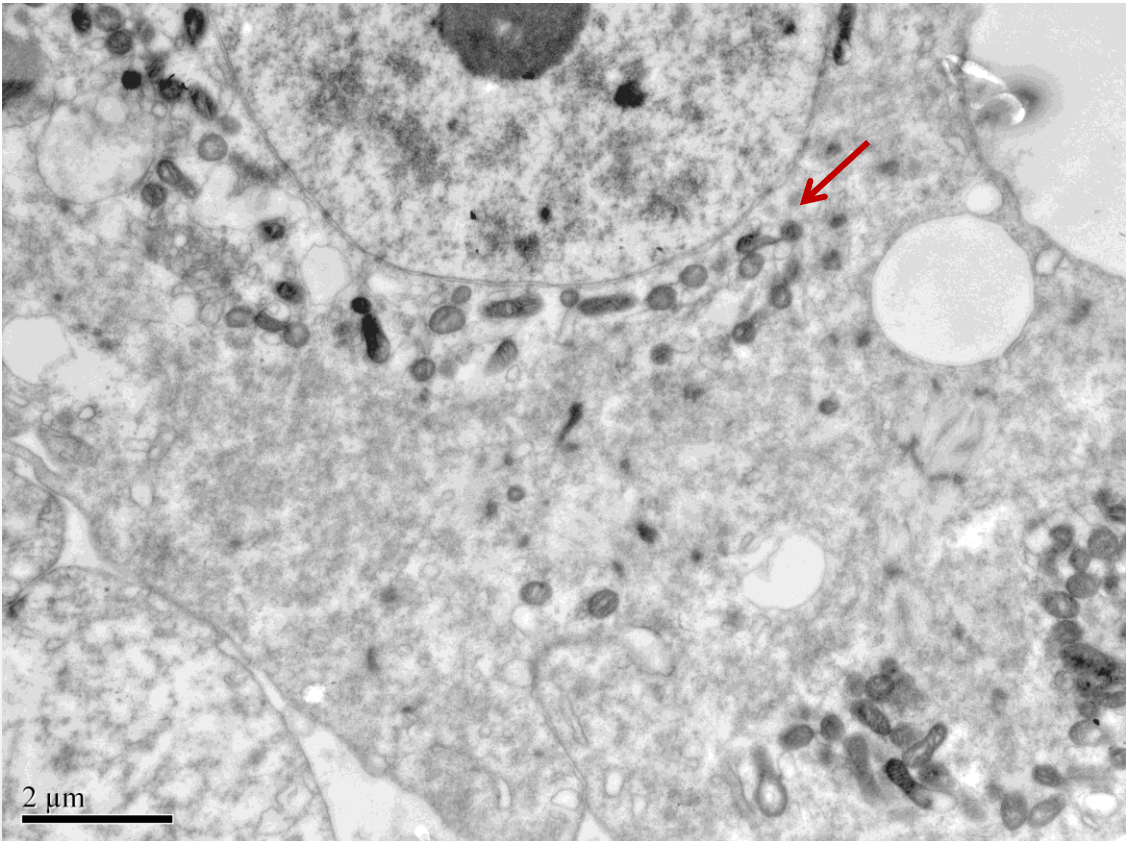

B

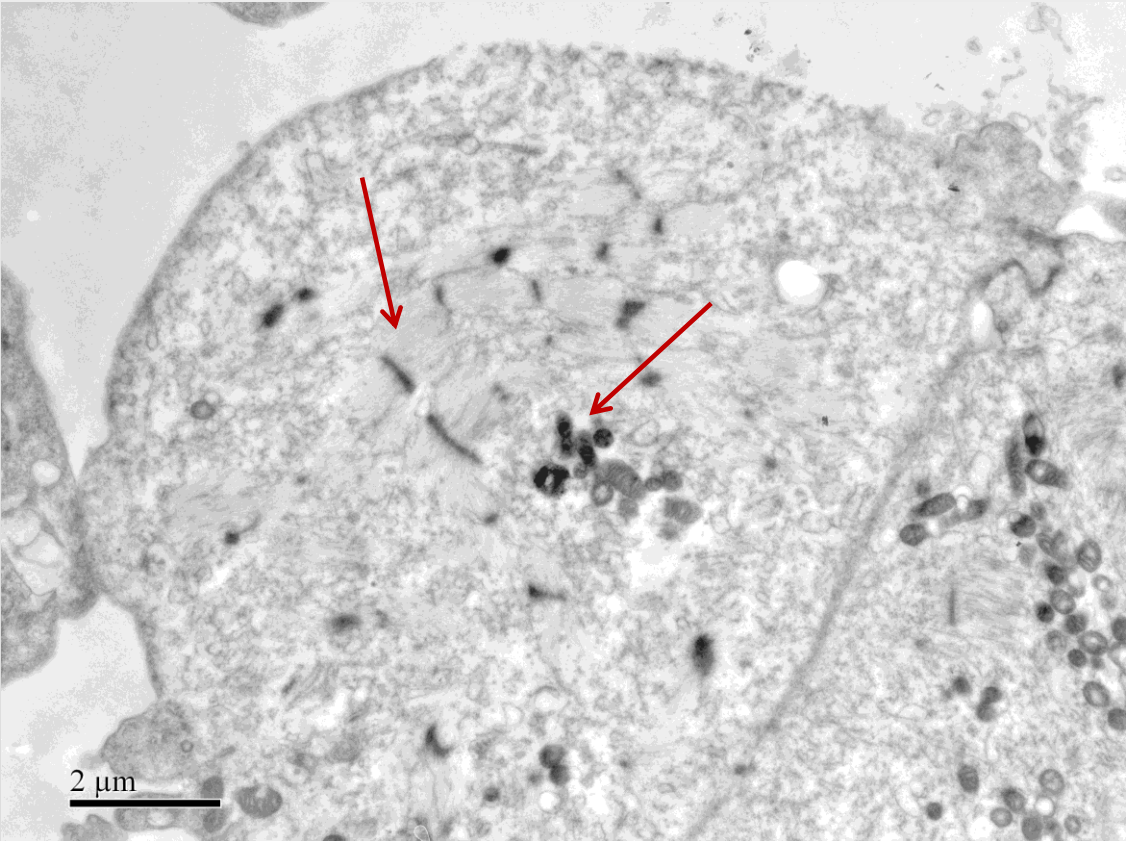

# Supplemental Figure 2

A

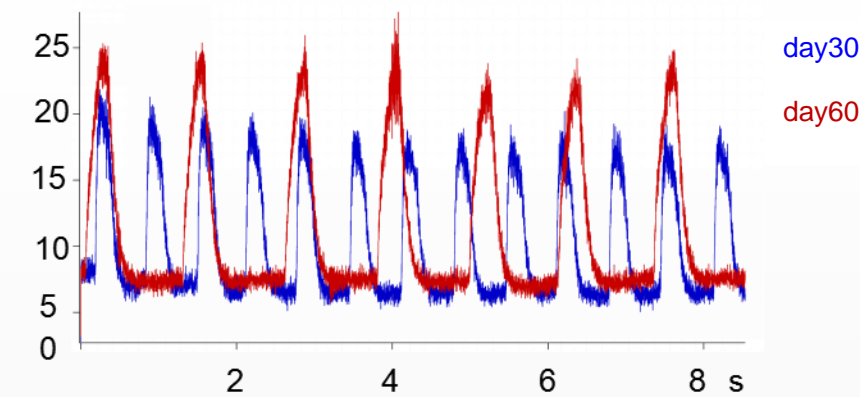

B

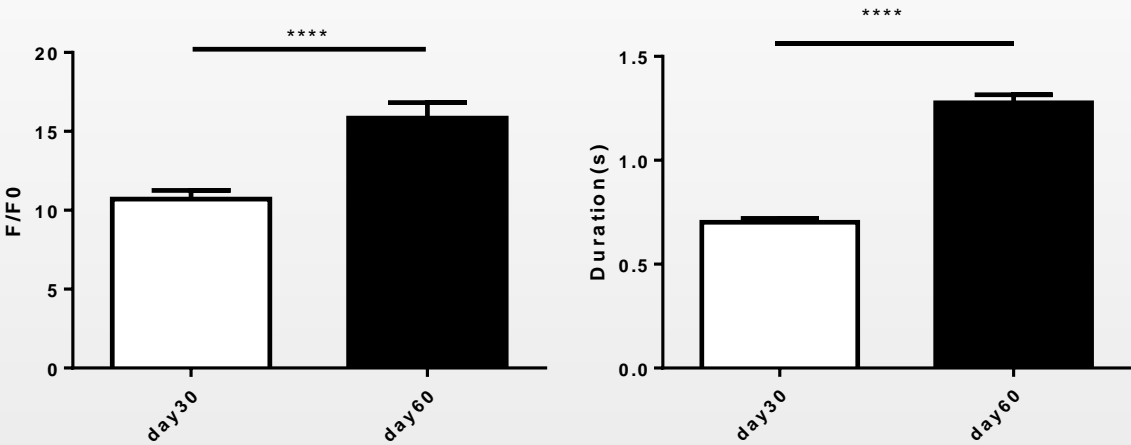

Supplement: Supplementary file 1 [file JCMM-23-4627-s001.pdf]
