# Supplementary material for: Doxorubicin‐induced cardiotoxicity is maturation dependent due to the shift from topoisomerase IIα to IIβ in human stem cell derived cardiomyocytes
Source: J Cell Mol Med. 2019 May 20;23(7):4627–39. doi: 10.1111/jcmm.14346 (PMC6584544; doi:10.1111/jcmm.14346)
Supplement: Supplementary file 2 [file JCMM-23-4627-s002.pdf]

Supplementary information, Table S1 Primers used for RT-PCRs

Gene name	Direction	Primer sequence
MYL2	Forward	TTCTCAACGCATTCAAGGTG
MYL2	Reverse	CTGGTCGATCTCCTCTTTGG
MYL7	Forward	CCGTCTTCCTCACGCTCTT
MYL7	Reverse	TGAACTCATCCTTGTTCACCAC
TNNT2	Forward	AGCATCTATAACTTGGAGGCAGAG
TNNT2	Reverse	TGGAGACTTTCTGGTTATCGTTG
LRRC1	Forward	CTGGGTACTCTTGTTCTCAG
LRRC1	Reverse	TCCCGTTCCTCTTCTTCATC
TNNI3	Forward	CTGCAGATTGCAAAGCAAGA
TNNI3	Reverse	CCTCCTTCTTCACCTGCTTG
MYH6	Forward	TCTCCGACAACGCCTATCAGTAC
MYH6	Reverse	GTCACCTATGGCTGCAATGCT
MYH7	Forward	GGCAAGACAGTGACCGTGAAG
MYH7	Reverse	CGTAGCGATCCTTGAGGTTGTA
CACNA1C	Forward	CAGAGGCTACGATTTGAGGA
CACNA1C	Reverse	GCTTCACAAAGAGGTCGTGT
KCNH2	Forward	AATCGCCTTCTACCGGAAAG
KCNH2	Reverse	CACCATGTCCTTCTCCATCAC
KCNJ2	Forward	AAGACGGTATGAAGTTGGCC
KCNJ2	Reverse	CGGGTGTGGACTTTACTCTTC
KCNQ1	Forward	TCTGTCTTTGCCATCTCCTTC
KCNQ1	Reverse	CCTCCATGCGGTCTGAATG
SCN5A	Forward	CTGACCTCACCATCACTATGTG
SCN5A	Reverse	GCTGTGAAAATCCCTGTGAAG
RYR2	Forward	AGAACTTACACACGCGACCTG
RYR2	Reverse	CATCTCTAACCGGACCATACTGC
SERCA2A	Forward	GATCACACCGCTGAATCTG

SERCA2A	Reverse	AGTATTGCGGGTTGTTCCAG
MKI67	Forward	TGACTTCCTTCCATTCTGAAGAC
MKI67	Reverse	TGGGTCTGTTATTGATGAGCC
BCL-2	Forward	GAGAAATCAAACAGAGGCCG
BCL-2	Reverse	CTGAGTACCTGAACCGGCA
BAX	Forward	AGCTTCTTGGTGGACGCAT
BAX	Reverse	CAGAGGCGGGGTTTCATC
TOP2A	Forward	TGCCAATGTAGTTTGTTTCTTTG
TOP2A	Reverse	GCCCTCAAGAAGATGGTGTG
TOP2B	Forward	TTGTTGGCAGTTTCTGACTCTT
TOP2B	Reverse	ATGGCCAAGTCGGGTGG
GAPD	Forward	AATGAAGGGGTCATTGATGG
GAPD	Reverse	AAGGTGAAGGTCGGAGTCAA
